# PD-1 and TIGIT blockade differentially affect tumour cell survival under hypoxia and glucose deprived conditions in oesophageal adenocarcinoma; implications for overcoming resistance to PD-1 blockade in hypoxic tumours

**DOI:** 10.1016/j.tranon.2022.101381

**Published:** 2022-03-01

**Authors:** Maria Davern, Marie-Claire Fitzgerald, Croí E. Buckley, Aisling B. Heeran, Noel E. Donlon, Jason McGrath, Fiona O’ Connell, Malvika R. Deshpande, Conall Hayes, Jamie MacDonald, Andrew D. Sheppard, John V. Reynolds, Stephen G. Maher, Niamh Lynam-Lennon, Brona Murphy, Joanne Lysaght

**Affiliations:** a Cancer Immunology and Immunotherapy Group, Department of Surgery, Trinity St. James's Cancer Institute, Trinity Translational Medicine Institute, St. James's Hospital campus, Dublin 8, Ireland; b Department of Physiology & Medical Physics, Royal College of Surgeons in Ireland, D02 YN77 Dublin 2, Ireland; c Translational Radiobiology and Diagnostics Group, Department of Surgery, Trinity St. James's Cancer Institute, Trinity Translational Medicine Institute, St. James's Hospital campus, Dublin 8, Ireland; d Translational Gastrointestinal Research Group, Department of Surgery, Trinity St. James's Cancer Institute, Trinity Translational Medicine Institute, St. James's Hospital campus, Dublin 8, Ireland; e Cancer Chemoradiation Group, Department of Surgery, Trinity St. James's Cancer Institute, Trinity Translational Medicine Institute, St. James's Hospital campus, Dublin 8, Ireland; f Department of Surgery, Trinity St. James's Cancer Institute, Trinity Translational Medicine Institute, St. James's Hospital, Trinity College Dublin, Dublin, Ireland

**Keywords:** Bcl-xl, GLUT1, Glycolysis, Immune checkpoints, Oxidative phosphorylation

## Abstract

Recent studies have demontrated that immune checkpoint receptors are expressed on the surface of oesophageal adenocarcinoma (OAC) cells and might confer a survival advantage. This study explores the role of PD-1 and TIGIT signalling in OAC cells in either promoting or inhibiting the survival of OAC cells under characteristic features of the tumour microenvironment including nutrient-deprivation and hypoxia. PD-1 and TIGIT are expressed in normal and pre-malignant oesophageal epithelial cells and this expression significantly decreases along the normal- Barrett's Oesophagus- OAC disease sequence. However, glucose-deprivation and hypoxia significantly upregulated PD-1 and TIGIT on the surface of OAC cells in vitro. PD-1 blockade decreased OAC cell proliferation under normoxia but enhanced proliferation and decreased cell death in OAC cells under hypoxia and glucose-deprivation. TIGIT blockade decreased proliferation and induced OAC cell death, an effect that was maintained under nutrient-deprivation and hypoxia. Basal respiration and glycolytic reserve were enhanced and GLUT1 was upregulated on the surface of a subpopulation of OAC cells following PD-1 blockade. In contrast, TIGIT blockade enhanced a glycolytic phenotype in OAC cells, yet decreased other metabolic parameters including oxidative phosphorylation and basal respiration. Interestingly, inhibition of oxidative phosphorylation significantly upregulated TIGIT expression and inhibition of oxidative phosphorylation and glycolysis significantly decreased PD-1 on the surface of a subpopulation of OAC cells in vitro. These findings suggest an immune-independent mechanism for PD-1 inhibitor resistance in hypoxic tumours and suggest that TIGIT might be a more effective therapeutic target in OAC compared with PD-1 for treating hypoxic tumours.

## Highlights

• PD-1 and TIGIT expression are highly expressed on the surface of oesophageal epithelial cells during the early stages of metaplasia.
• Glucose deprivation and hypoxia upregulate PD-1 and TIGIT on the surface of oesophageal adenocarcinoma (OAC) cells in vitro.
• PD-1 and TIGIT blockade decrease Bcl-2 and Bcl-xL expression in OAC cells.
• PD-1 blockade in OAC cells enhances basal respiration and glycolytic reserve and upregulates GLUT1 on the surface of a subpopulation of OAC cells.
• PD-1 inhibition confers a survival advantage to OAC cells under glucose deprivation and hypoxia.
• TIGIT blockade decreases OAC cell proliferation and induces OAC cell death under normoxia, hypoxia and nutrient deprivation.
• TIGIT blockade increases ECAR yet decreases a range of metabolic parameters in OAC cells.

## Introduction

Targeting inhibitory immune checkpoints (ICs) is an attractive therapeutic strategy to reinvigorate exhausted anti-tumour immunity in oeosphageal adenocarcinoma (OAC). Interestingly, recent studies have demonstrated that IC ligands and receptors are expressed on the surface of tumour cells in a range of malignancies such as melanoma, lung, head and neck and colorectal cancer [1], [2], [3], [4], [5], [6], [7], [8], [9]. In addition IC tumour cell intrinsic signalling has been shown to promote various hallmarks of cancer including glycolysis [10], DNA repair [11], proliferation [1] invasion and migration [12,13].

We have previously shown that blockade of PD-1 and PD-L1 tumour cell intrinsic signalling in OAC cells enhanced the cytotoxicity of the FLOT chemotherapy regimen in vitro [14]. Similarly, Liu et al., have demonstrated that PD-1 intrinsic signalling in gastric tumour cells protects against 5-FU induced cell death [15]. However, it remains to be investigated if IC-intrinsic signalling in OAC cells might confer a survival advantage under the harsh physiological conditions of the tumour microenvironment (TME). Therefore, we investigated the effect of characteristic features of the hostile TME, such as nutrient deprivation and hypoxia on IC expression on OAC cells. Importantly, we assessed if immune checkpoint blockade (ICB) under these TME conditions might promote or inhibit the surival of OAC cells. The effect of ICB on the metabolic phenotype of OAC cells was also assessed as nutrient deprivation and hypoxia have profound effects on metabolism in OAC cells [16]. Furthermore, activation of PD-L1 signalling in renal cells [17] and non-small cell lung cancer cells [18] increases glycolysis in those tumour cells.

Importantly, this current study also investigated the expression of a range of inhibitory ICs on the surface of oesophageal cells along the normal-Barrett's Oesophagus-OAC disease sequence to determine if expression is altered as disease progresses. Barrett's oesophagus (BO) is a pathologically defined precursor of OAC and is characterised by specialised intestinal metaplasia, which develops as a consequence of long-term reflux of acid and bile [19]. The malignant progression potential of BO to OAC is approximately 0.12% per annum [19]. Previous studies have demonstrated that TIM-3^5^ and PD-1^20^ IC receptors were significantly upregulated along the normal-pre-malignant-carcinoma disease sequence in cervical cancer and pancreatic cancer, respectively. Therefore, gaining a deeper insight into the changes in IC expression across the malignant progression sequence could identify appropriate ICs to target in the pre-malignant and malignant setting to improve clinical outcomes for patients.

## Methods

### Human normal, BO and OAC datasets

Normalized mRNA expression and associated metadata data were obtained and assessed from the Broad Institute, The Cancer Genome Atlas (TCGA) study of normal oesophageal epithelium, Barrett's metaplasia, and oesophageal adenocarcinomas as previously described [21]. RNA-sequenced data was only available for PD-L2 and CD160 IC ligands and PD-1, LAG-3 and A2aR IC receptors across the normal-BO—OAC disease sequence. RNA-sequenced data was not available for PD-L1, TIGIT and TIM-3 in the online dataset.

### Culture of cell lines

Human normal oesophageal cells (HET-1A), BO cells (QH), OAC cells (OE33 and OE19 cells) were purchased from European Collection of Cell Cultures. HET-1A cells were grown in flasks pre-coated with a solution of fibronectin (0.01 mg/ml, Merck, Germany), collagen type I (0.03 mg/ml, Corning, USA) and bovine serum albumin (0.01 mg/ml, Sigma, USA) dissolved in PBS and cultured in serum free Bronchia/Trachea Epithelial Cell Growth Medium (511–500, Merck, Germany). HET-1A cells were detached from flask using accutase (Sigma, USA) diluted 1:15 in PBS. QH cells were grown in Bronchia/Trachea Epithelial Cell Growth Medium supplemented with 1% (v/v) penicillin-streptomycin (50 U/ml penicillin 100 μg/ml streptomycin) and 10% (v/v) foetal bovine serum (ThermoFisher Scientific, Ireland) and detached from flask using trypsin-EDTA solution (Sigma, USA). OE33 cells and OE19 cells were grown in RPMI 1640 medium with 2 mM l-glutamine (ThermoFisher Scientific, Ireland) and supplemented with 1% (v/v) penicillin-streptomycin (50 U/ml penicillin 100 μg/ml streptomycin (P/S)) and 10% (v/v) foetal bovine serum (FBS) (ThermoFisher Scientific, Ireland) and detached using trypsin-EDTA solution. All cell lines were maintained in a humidified chamber at 37 °C 5% CO_2_ and were tested regularly to ensure mycoplasma negativity.

### Nutrient deprivation and hypoxia treatment

OE33 cells and OE19 cells were cultured in complete RPMI (cRPMI, 10% FBS, 1% P/S), serum-free RPMI (0% FBS, 1% P/S), glucose-free RPMI (Gibco (11560406), 10% FBS, 1% P/S), dual glucose-free and serum deprived RPMI (Gibco (11560406), 0% FBS, 1% P/S) under normoxic conditions (37 °C, 5% CO_2,_ 21% atmospheric O_2_) or hypoxic conditions (37 °C, 5% CO_2,_ 0.5% O_2_) using the H35 Don Whitley hypoxia station.

### Flow cytometry staining

OE33 or OE19 cells were cultured under nutrient-deprivation or hypoxic conditions or a combination of both for 24 h or 48 h or treated with 2-deoxy-d-glucose (2DG) or oligomycin for 24 h. Cells were trypsinised and stained with zombie aqua viability dye (Biolegend, USA) for gating on live cells and subsequently stained with PD-1-PE/Cy7 or TIGIT-PE/Cy7 (Biolegend, USA) antibodies. OE33 or OE19 cells were treated with PD-1 blockade or TIGIT blockade for 24 h (Pembrolizumab (10 μg/ml) or anti-TIGIT monoclonal antibody from Biolgend, USA (10 μg/ml)). OAC cell lines were trypsinised and subsequently stained with zombie aqua viability dye (Biolgend, USA) and GLUT1-AF647 antibody (BD Biosciences, USA). Cells were fixed with 1% paraformaldehyde solution and acquired using BD FACs CANTO II (BD Biosciences) using Diva software and analysed using FlowJo v10 software (TreeStar Inc.).

### BrdU assay

A BrdU assay (Sigma, USA) was used to assess the effect of TIGIT blockade or PD-1 blockade on the proliferation rate of OE33 cells and OE19 cells. Cells were seeded at 5 × 10^3^ in 100 μl/ well in a flat 96 well plate in complete RPMI (10% FBS) and were allowed adhere overnight at 37  °C, 5% CO_2_. The media was removed, and cells were cultured for 24 h in complete RPMI or nutrient deprived media or under hypoxia in the absence or presence of PD-1 blockade or TIGIT blockade. The cell proliferation was then assessed using a BrdU cell proliferation ELISA (Roche Diagnostics Ltd., Sussex, UK) according to the manufacturer's guidelines. The optical density at 450 nm and 690 nm (reference wavelength) was measured using the Versa Max microplate reader (Molecular Devices, Sunnyvale, CA, USA) to determine a viable cell number. Wells containing cells but no BrdU label were used to subtract the background absorbances and the percentage increase/decrease in proliferation was calculated relative to the untreated cells. All the data were analysed from three independent experiments.

### Annexin V and propidium iodide assay

Apoptosis was measured using annexin V (AV)-FITC and propidium iodide (PI) staining and was assessed by flow cytometry. OE33 cells and OE19 cells were cultured under serum deprivation (0% foetal bovine serum (FBS)), glucose deprivation (0% glucose, 10% FBS), dual glucose-serum deprivation (0% FBS, 0% glucose), hypoxia (0.5% O_2_), dual serum deprived-hypoxic conditions, dual glucose deprived-hypoxic conditions and dual serum-glucose deprived-hypoxic conditions in the presence or absence of anti-PD-1 Pembrolizumab, (10 μg/ml) or anti-TIGIT monoclonal antibody (10 μg/ml)) for 24 h. Cells were stained with Annexin V-FITC (Biolegend, USA) and 1:4000 PI (Invitrogen, Carlsbad, CA, USA), and samples were acquired using BD FACs CANTO II (BD Biosciences) using Diva software and analysed using FlowJo v10 software (TreeStar, Inc., Ashland, Oregon).

### Western blot analysis

OE33 and OE19 cells were homogenized in RIPA lysis buffer (150 mM NaCl, 0.1% Triton X-100, 0.5% sodium deoxycholate, 0.1% sodium dodecyl sulfate, 50 mM Tris), with added protease/phosphatase inhibitor cocktails (Sigma-Aldrich, Arklow, Ireland). Protein concentration of samples was determined using a BCA protein assay (Pierce, Rockford, IL, USA), and 20 μg samples were boiled in Laemmli buffer and separated on 12% SDS-PAGE gels. Proteins were transferred to nitrocellulose membranes using wet transfer. The membranes were blocked in 5% non-fat milk TBS, 0.1% Tween 20 detergent (TBST) for 1 h at room temperature prior to being incubated with primary antibodies overnight at 4 °C. The following primary antibodies were used: rabbit anti-Bcl-xL (1:1000) (Cat #2764S, Cell Signalling, Danvers, MA, US); mouse anti-Bcl-2 (1:500) (Cat #sc-7382, Santa Cruz Biotechnology, Santa Cruz, CA, US); mouse anti-GAPDH (1:1000) (Cat #MAB374, Sigma-Aldrich) mouse anti-β-actin (1:1000) (Cat #A5441, Sigma-Aldrich). Membranes were then washed three times with TBST for 5 min prior to being incubated with goat anti-mouse IgG (1:15,000) (#AP124P, Merck KGaA, Darmstadt, Germany) or goat anti-rabbit IgG (1:5000) (#AP132P, Merck KGaA, Darmstadt, Germany) peroxidase-conjugated secondary antibodies for 1 h at room temperature. Protein bands were visualized using the Immobilon western chemiluminescent HRP substrate (Millipore Sigma) and images were captured using a LAS-3000 imager equipped with a cooled 12 bit digital CCD camera (Fujifilm UK Ltd, Bedfordshire, UK). To guarantee accurate quantifications, special care was taken not to over-expose the protein bands. Densitometry was carried out on 12-bit raw images using Image Studio Lite v5.2 (LI-COR Biosciences Ltd., UK).

### Real-time metabolic analysis

OE33 and OE19 cells were seeded in five wells per treatment group at a density of 10,000 cells/well, in 24-well cell culture XFe24 microplates (Agilent Technologies, Santa Clara, CA, USA) at a volume of 100 μL and allowed to adhere at 37 °C in 5% CO2/95% air. Five hours later, an additional 150 μL/well complete cell culture RPMI medium was added. Following 48 h of incubation, media was removed and cells were washed with unbuffered Dulbecco's Modified Eagle's medium (DMEM) supplemented with 10 mM of glucose and 10 mM of sodium pyruvate, (pH 7.4) and incubated for one hour at 37 °C in a CO2-free incubator. The oxygen consumption rate (OCR) and extracellular acidification rate (ECAR) were measured using a Seahorse Biosciences XFe24 Extracellular Flux Analyser (Agilent Technologies, Santa Clara, CA, USA). Three basal measurements of OCR and ECAR were taken over 24 min consisting of three repeats of mix (three min)/wait (2 min)/measurement (3 min) to establish basal respiration. Three additional measurements were obtained following the injection of three mitochondrial inhibitors including oligomycin (Sigma Aldrich, Missouri, USA), antimycin-A (Sigma Aldrich, Missouri, USA) and an uncoupling agent Carbonyl cyanide 4-(trifluoromethoxy) phenylhydrazone (FCCP) (Sigma Aldrich, Missouri, USA). ATP turnover was calculated by subtracting the OCR post oligomycin injection from baseline OCR prior to oligomycin addition. Proton leak was calculated by subtracting OCR post antimycin-A addition from OCR post oligomycin addition. Maximal respiration was calculated by subtracting OCR post antimycin addition from OCR post FCCP addition. Non-mitochondrial respiration was determined as the OCR value post antimycin-A addition. All the measurements were normalised to cell number using the crystal violet assay, transferring the eluted stain to a 96-well plate before reading.

### Crystal violet

Cells were fixed with 1% glutaraldehyde (Sigma-Aldrich, Missouri, USA) for 15 min at room temperature. The fixative was removed, and cells were washed with PBS and stained with 0.1% crystal violet for 30 min at room temperature. Plates were left to air dry and incubated with 50 µL of 1% Triton X-100 in PBS on a plate shaker for 30 min at room temperature. absorbance was read at 595 nm on a VersaMax microplate reader (Molecular Devices, Sunnyvale, CA, USA).

### Statistical analysis

Data were analysed using GraphPad Prism (GraphPad Prism, San Diego, CA, USA) and was expressed as mean ± SEM. Statistical differences between two treatments in a particular cell line were analysed using a paired parametric Student's *t*-test. To compare the statistical differences between two different cell lines an unpaired parametric *t*-test was conducted. To compare differences between patients and different treatments an unpaired non-parametric *t*-test was conducted. Statistical significance was determined as *p* ≤ 0.05.

## Results

### PD-1 and TIGIT expression on the surface of oesophageal epithelial cells decreases along the normal-BO—OAC disease sequence

RNA-sequenced data of normal, BO and OAC tissue samples available from online datasets was profiled for IC expression to provide a holistic view of the alterations or lack of alterations in the overall IC expression profile within the tissue samples. These samples encapsulate all the cell types including immune cells, non-cancer epithelial cells, BO cells and OAC cells in the tissue samples taken from normal, pre-neoplastic and neoplastic tissue oesophageal tissue. The rationale was to provide a broader picture on the expression landscape of a wide range of ICs across the disease sequence in OAC.

**Fig. 1:**
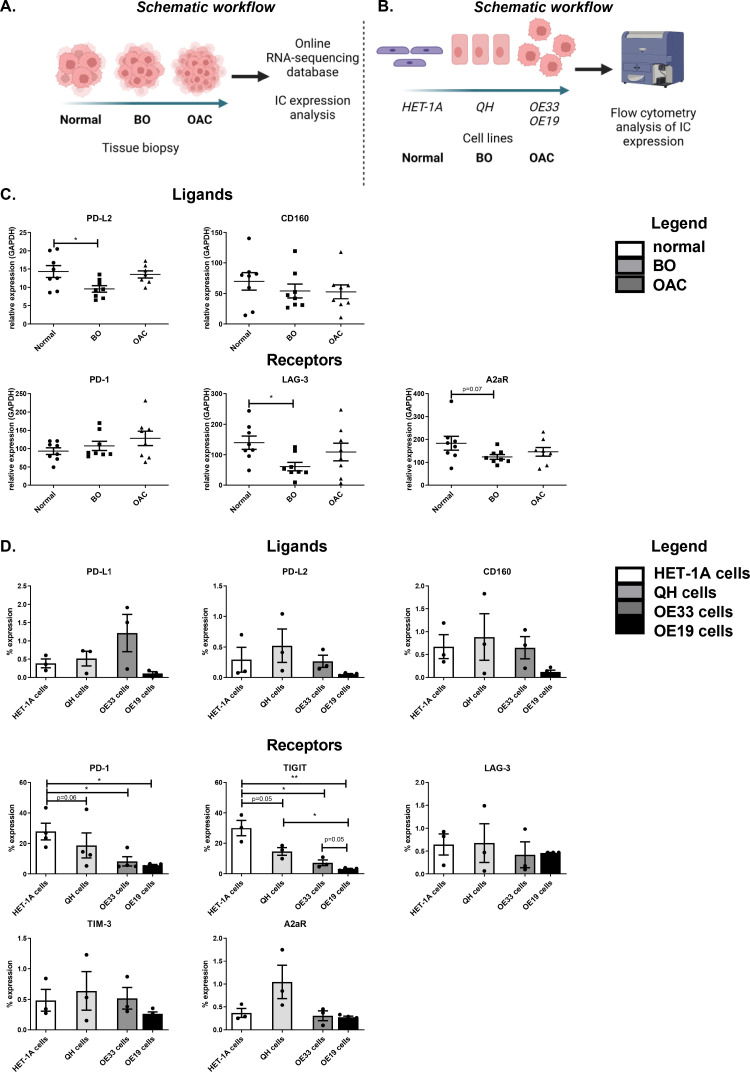
The expression of PD-1 and TIGIT on the surface of oesophageal epithelial cells decreases along the normal-BO—OAC disease sequence. (A) Schematic workflow for assessing mRNA levels of inhibitory IC ligands and receptors were assessed in 8 normal oesophageal tissue samples, 8 BO tissue samples and 8 OAC tissue samples from online RNA-sequenced databases [21]. The inhibitory IC ligands available in the database included: PD-L2 and CD160 and the IC receptors available included: PD-1, LAG-3 and A2aR (C). Kruskal-Wallis with uncorrected Dunn's test. Expression presented relative to GAPDH ± SEM. (B) Schematic workflow for analysing the IC ligand and IC receptor expression on the surface of HET-1A, QH, OE33 and OE19 cells by flow cytometry. The inhibitory ligands assessed include PD-L1, PD-L2 and CD160 and the inhibitory IC receptors include PD-1, TIGIT, LAG-3, TIM-3 and A2aR (D). Pooled analysis of 4 independent biological replicates that were carried out using singlet technical replicates. Mixed-effects analysis, Tukey's multiple comparison. *p<0.05 and **p<0.01. Data expressed as ± SEM.

However, a caveat of the RNA-seq analysis (Fig. 1**A.**) included the inability to distinguish between ICs that were expressed on the epithelial cells or on infiltrating immune cells across these different tissue types. Therefore, we also assessed IC expression profiles on epithelial cell lines representing normal oesophageal epithelial cells, BO epithelial cells and OAC epithelial cells. This experimental approach was utilised to discern if IC expression profiles are altered on epithelial cells specifically but also within the overall tissue sample across the normal-pre-neoplastic-neoplastic disease sequence.

RNA-sequenced data was only available for PD-L2 and CD160 IC ligands and PD-1, LAG-3 and A2aR IC receptors across the normal-BO—OAC disease sequence (Fig. 1**C.**). RNA-sequenced data was not available for PD-L1, TIGIT and TIM-3 in the online dataset.

We observed only modest changes in IC expression across the disease sequence. Levels of PD-L2 mRNA were significantly reduced in BO tissue compared with normal oesophageal tissue and trended toward a significant increase in the mRNA levels of PD-L2 in OAC tissue compared with BO tissue (normal:14.31 ± 1.6% vs. BO:9.6 ± 0.9%, OAC:13.53 ± 0.9% *p* = 0.02 and *p* = 0.06, respectively) (Fig. 1**C.**). In addition, mRNA levels of LAG-3 were significantly reduced in BO tissue compared with normal oesophageal tissue (normal:139.3 ± 21.6% vs. BO:61.38 ± 13.7%, *p* = 0.01), (Fig. 1**C.**). There were no significant alterations in A2aR, CD160 or PD-1 across the disease sequence at the mRNA level (Fig. 1**C.**).

To determine if IC expression profiles on just the oesophageal epithelial cells was altered across the normal-BO—OAC disease sequence, cell line models of normal oesophageal epithelial cells (HET-1A cells), metaplastic BO cells (QH cells) and OAC cells (OE33 cells and OE19 cells) were screened by flow cytometry for the surface expression of a range of ICs (Fig. 1**D.**). Interestingly, the surface expression of PD-1 on oesophageal epithelial cells significantly decreased across the normal-BO—OAC disease sequence. OE33 cells expressed significantly less PD-1 compared with the HET-1A cells (8.30 ± 3.0% vs. 27.8 ± 5.5%, *p* = 0.02) (Fig. 1**D.**). Similar trends were observed in the OE19 cells, in which PD-1 was expressed at significantly lower levels on OE19 cells compared with the HET-1A cells (5.80 ± 0.5% vs. 27.8 ± 5.5%, *p* = 0.008) (Fig. 1**D.**). Similarly, TIGIT protein expression significantly decreased on the surface of epithelial cells across the normal-BO—OAC disease sequence with a reduction in TIGIT expression on the surface of QH cells compared with HET-1A cells (14.69 ± 2.5% vs. 30.00 ± 5.1%, *p* = 0.05). A significantly lower percentage of OE33 cells expressed TIGIT compared with the HET-1A cells (7.29 ± 1.9% vs. 30.00 ± 5.1%, *p* = 0.01), as was the case for OE19 cells compared with HET-1A cells (3.20 ± 0.4% vs. 30.00 ± 5.1%, *p* = 0.001) (Fig. 1**D.**). Furthermore, significantly less OE19 cells expressed TIGIT compared with QH cells (3.20 ± 0.4% vs. 14.69 ± 2.5%, *p* = 0.02). Low levels of the IC ligands PD-L1, PD-L2, CD160, LAG-3, TIM-3 and A2aR were detected on the surface of HET-1A cells, QH cells, OE33 cells and OE19 cells, however, there was no significant difference in the expression of these ICs between the different cell types across the normal-BO—OAC disease sequence (Fig. 1**D.**).

Overall, these findings demonstrated that IC expression is modestly but significantly altered in oesophageal tissue across the normal-BO—OAC disease sequence at the mRNA level. Reductions in PD-L2 and LAG-3 mRNA were found specifically in BO tissue compared with normal tissue however, the levels of PD-L2 and LAG-3 mRNA were comparable between normal tissue and OAC tissue. Interestingly, specific alterations in IC expression profiles on the surface of epithelial cells was observed, in which PD-1 and TIGIT were significantly decreased across the disease sequence from normal-BO—OAC.

### Glucose deprivation and hypoxia upregulate PD-1 and TIGIT on the surface of OAC cells

**Fig. 2:**
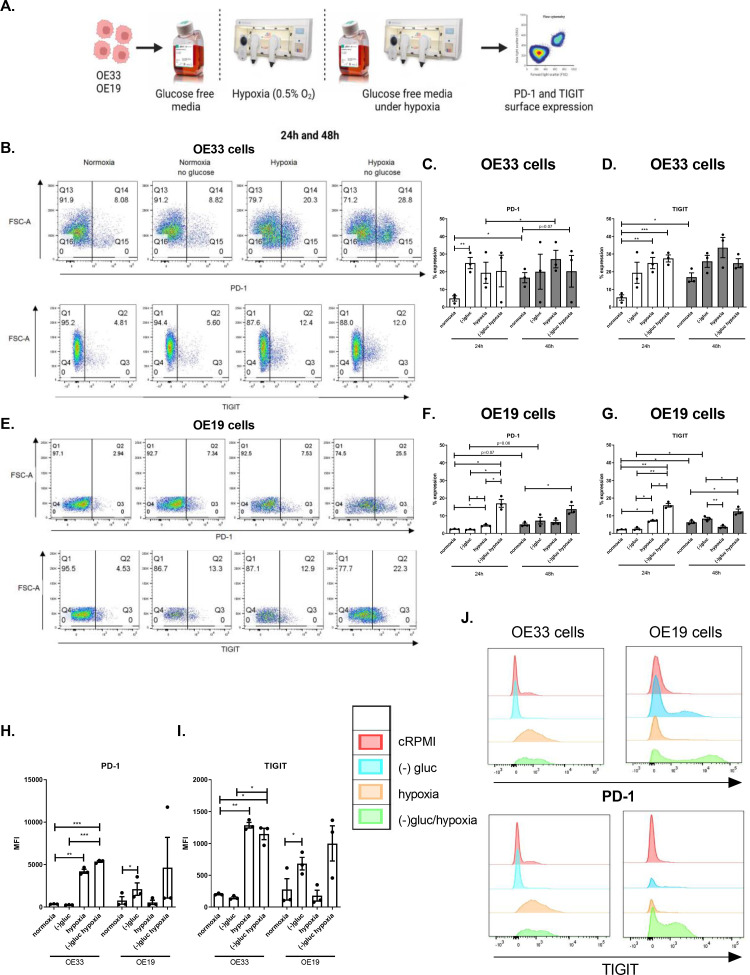
Glucose deprivation and hypoxia significantly increase the percentage of viable OAC cells expressing PD-1 and TIGIT. (A) Schematic workflow. OE33 and OE19 cells were cultured in normoxia (white bars), with glucose deprivation, hypoxia (0.5% O_2_) (grey bars) and combined glucose deprived hypoxic conditions for 24 h and 48 h. The surface expression of inhibitory IC receptors PD-1 and TIGIT on viable OAC cells was assessed by flow cytometry. Representative dot plots displayed showing effect of each condition following 24 h treatment on PD-1 and TIGIT expression on the surface of viable OE33 cells (B) and OE19 cells (E). (C) and (D) depicts PD-1 and TIGIT percentage expression on OE33 cells and (F) and (G) depicts PD-1 and TIGIT percentage expression on OE19 cells. Median fluorescence intensity (MFI) for PD-1 (H) and TIGIT (I) expression in OE33 and OE19 cells following 24 h under each condition. (J) Representative histograms showing MFI levels for PD-1 and TIGIT in OE33 and OE19 cells under each condition. Pooled analysis for 3 independent biological replicates carried out using singlet technical replicates. *p<0.05, **p<0.01 and ***p<0.001. Paired, parametric *t*-test. Data expressed as ± SEM.

As OAC cells express significantly less PD-1 and TIGIT compared with normal oesophageal cells, we sought to investigate if features of the hostile TME including hypoxia and nutrient deprivation might alter the expression of PD-1 and TIGIT on the surface of viable OAC cells in vitro (Fig. 2**.**). 24 h glucose deprivation significantly upregulated PD-1 on the surface of viable OE33 cells compared with cells cultured in cRPMI in vitro (cRPMI:4.86 ± 1.5% vs. glucose-deprivation:24.9 ± 3.2%, *p* = 0.008), (Fig. 2**C.**). In addition, 24 h hypoxia and combined glucose-deprivation with hypoxia significantly upregulated PD-1 on the surface of viable OE19 cells in vitro (cRPMI:2.46 ± 0.1% vs. hypoxia:4.63 ± 0.4%, combined glucose-deprivation with hypoxia:17.03 ± 2.2%, *p* = 0.04 and *p* = 0.01, respectively), (Fig. 2**F.**). Similarly, 48 h combined glucose-deprivation with hypoxia significantly upregulated PD-1 on the surface of viable OE19 cells compared with cells cultured in cRPMI (cRPMI:5.18 ± 0.7% vs. combined glucose-deprivation with hypoxia:13.77 ± 2.0%, *p* = 0.02), (Fig. 2**F.**).

Furthermore, 24 h glucose deprivation and combined glucose-deprivation with hypoxia significantly upregulated TIGIT on the surface of viable OE33 cells compared with cells cultured in cRPMI in vitro (cRPMI:5.43 ± 1.6% vs. hypoxia:24.9 ± 3.2, combined glucose-deprivation with hypoxia:27.50 ± 2.0%, *p* = 0.007 and *p* = 0.003, respectively), (Fig. 2**D.**). Similarly, 24 h hypoxia and combined glucose-deprivation with hypoxia significantly upregulated TIGIT on the surface of viable OE19 cells compared with cells cultured in cRPMI in vitro (cRPMI:2.2 ± 0.1% vs. hypoxia:7.23 ± 0.5%, combined glucose-deprivation with hypoxia:15.93 ± 1.0%, *p* = 0.001 and *p* = 0005, respectively), (Fig. 2**G.**). Similarly, 48 h combined glucose-deprivation with hypoxia significantly upregulated TIGIT on the surface of viable OE19 cells compared with cells cultured in cRPMI in vitro (cRPMI:6.38 ± 0.7% vs. combined glucose-deprivation with hypoxia:12.50 ± 1.3%, *p* = 0.01), (Fig. 2**G.**).

Overall, glucose-deprivation, hypoxia, or combined glucose-deprivation with hypoxia significantly upregulated PD-1 and TIGIT on the surface of OAC cells in vitro.

### PD-1 blockade increases OAC cell proliferation under hypoxia and tigit blockade reduces OAC cell proliferation under dual glucose and serum deprivation

**Fig. 3:**
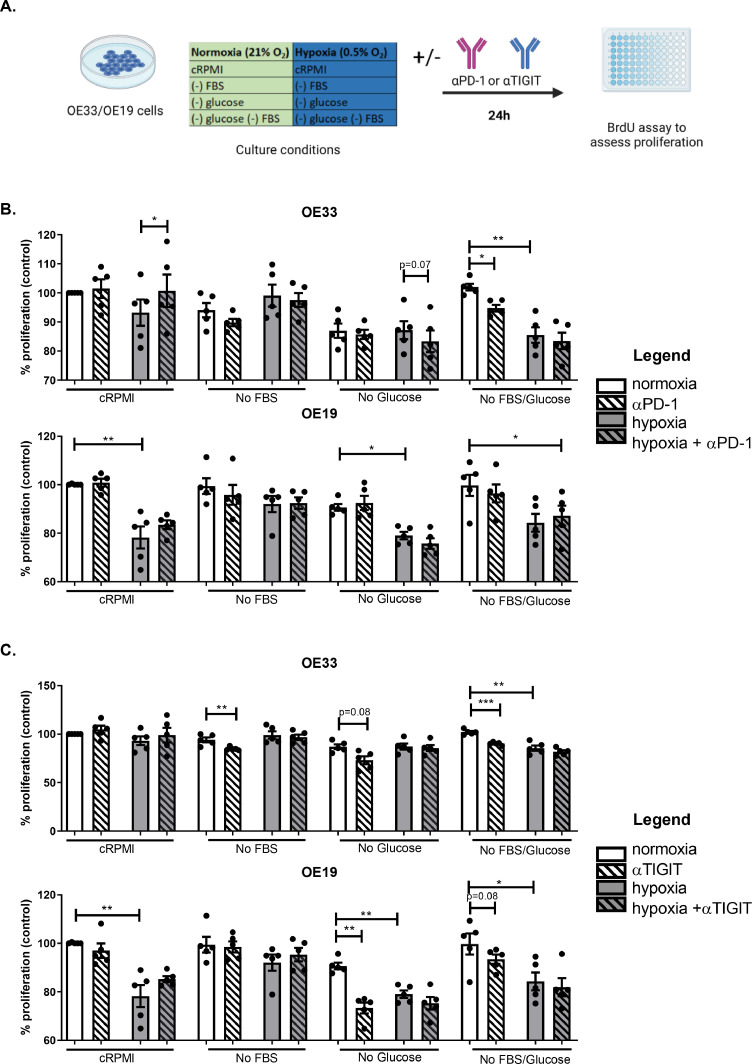
PD-1 blockade increases OAC cell proliferation under hypoxia whereas, TIGIT blockade decreases proliferation in OAC cells under glucose deprivation and serum deprivation. (A) Schematic workflow. OE33 and OE19 cells were cultured under cRPMI (complete media), serum deprivation (no FBS), glucose deprivation, cRPMI under hypoxia and combined serum deprivation-hypoxia and combined glucose deprivation-hypoxia for 24 in the absence or presence of αPD-1 (pembrolizumab, 10 μg/ml) (B) or αTIGIT monoclonal antibody (C). Pooled analysis for 5 independent biological replicates carried out in duplicate technical replicates were used for both the OE33 and OE19 cell line. A BrdU assay was used to determine the percentage of proliferating cells. Paired parametric *t*-test *p<0.05 and **p<0.01 and ***p<0.001. Data expressed as ± SEM.

Given that hostile features of the TME such as hypoxia and nutrient deprivation upregulated PD-1 and TIGIT on the surface of OAC cells, we aimed to investigate if PD-1 or TIGIT upregulation might provide OAC cells with a survival advantage under these unfavourable conditions. Therefore, the effect of single agent PD-1 blockade and TIGIT blockade on the viability of OAC cells was assessed under complete RPMI conditions, nutrient deprivation, hypoxia and combined nutrient deprivation-hypoxia following 24 h culture (Fig. 3**B. and** Fig. 3**C.,** respectively).

PD-1 blockade decreased the proliferation of OE33 cells under dual serum deprivation-glucose deprivation compared with untreated cells cultured in dual serum deprivation-glucose deprivation (84.8 ± 1.1% vs. 102.0 ± 1.2%, respectively *p* = 0.02), (Fig. 3**B.**). Contrastingly, PD-1 blockade increased the proliferation of OE33 cells under hypoxia compared with untreated cells cultured under hypoxia (100.7 ± 5.6% vs. 93.21 ± 4.5%, *p* = 0.04), (Fig. 3**B.**). PD-1 blockade did not have a significant effect on the proliferation of OE19 cells cultured in complete RPMI conditions, nutrient deprivation, hypoxia, or dual nutrient deprivation-hypoxia (Fig. 3**B.**).

Interestingly, TIGIT blockade significantly decreased the proliferation of OE33 cells under serum deprivation compared with untreated cells cultured in serum deprived media (84.84 ± 0.8% vs. 94.06 ± 2.4%, *p* = 0.008), (Fig. 3**C.**). Similarly, TIGIT blockade significantly decreased the proliferation of OE33 cells under dual serum-glucose deprivation compared with untreated cells cultured in dual serum-glucose deprivation (90.25 ± 0.7% vs. 102.0 ± 1.2%, *p* = 0.0007), (Fig. 3**C.**). In addition, TIGIT blockade significantly reduced OE19 cell proliferation under glucose deprivation compared with untreated cells cultured under glucose deprivation (73.33 ± 2.3% vs. 90.68 ± 1.4%, *p* = 0.002), (Fig. 3**C.**). TIGIT blockade did not significantly affect OE33 or OE19 cell proliferation under hypoxia or combined nutrient deprivation-hypoxic conditions (Fig. 3**C.**).

Overall, TIGIT blockade had the greatest effect in reducing OAC cell proliferation under glucose deprivation and serum deprivation. PD-1 blockade had similar effects in reducing proliferation of OE33 cells under dual serum-glucose deprivation however, PD-1 blockade increased OE33 cell proliferation under hypoxia.

### PD-1 blockade decreases whereas TIGIT blockade increases OAC cell death under hypoxia

To further interrogate the effects of PD-1 blockade and TIGIT blockade on OAC cell viability under nutrient deprivation and hypoxia, OE33 and OE19 cells were treated with PD-1 and TIGIT immune checkpoint inhibitors and OAC cell death was assessed by AV PI assay.

**Fig. 4:**
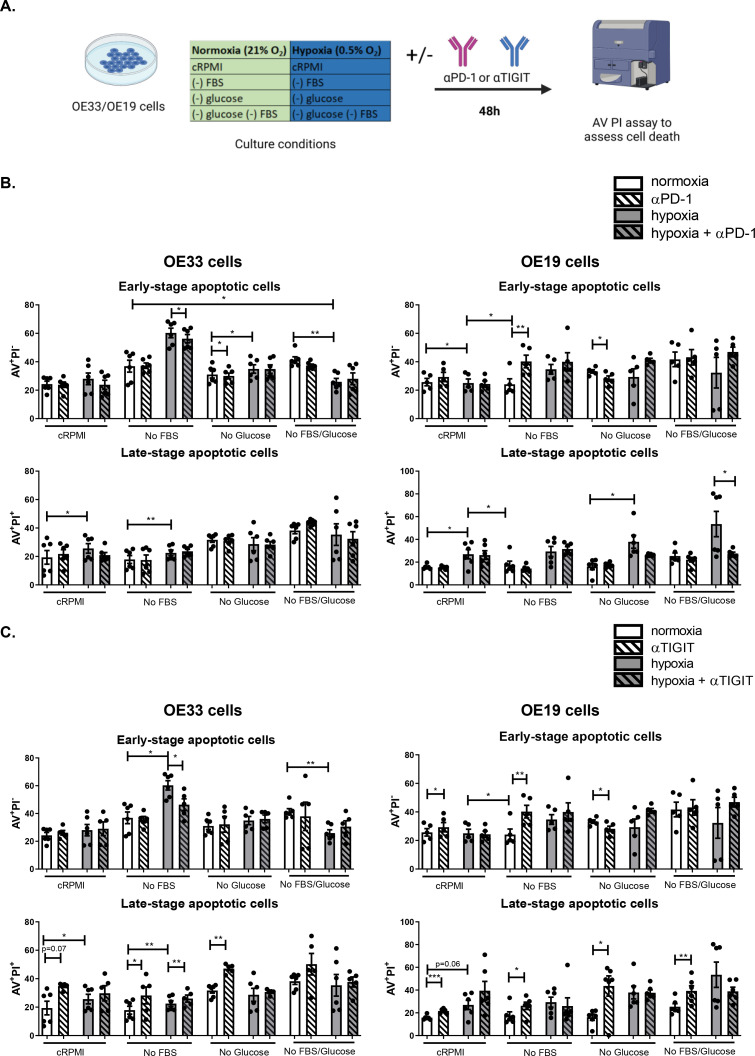
PD-1 blockade decreases OAC cell death under hypoxia and TIGIT blockade induces OAC cell death basally and under nutrient deprivation. (A) Schematic workflow. OE33 cells and OE19 cells were cultured under in complete media (cRPMI), serum deprived (no FBS), glucose deprived, combined serum deprived-glucose deprived, hypoxia and combined serum deprived-hypoxia, combined glucose deprived-hypoxia and combined serum deprived-glucose deprived-hypoxia for 48 h in the absence or presence of αPD-1 (B) or αTIGIT monoclonal antibody (C). Viability was determined by annexin V propidium iodide assay by flow cytometry. Viable cells (AV^−^PI^−^), early-stage apoptotic cells (AV^+^PI^−^), late-stage apoptotic cells (AV^+^PI^+^) and necrotic cells (AV^−^PI^+^) were characterised, only data for early-stage apoptotic and late-stage apoptotic cells shown. Pooled analysis for 5 independent biological replicates that were carried out in singlet. White bars denote normoxic conditions, grey bars denote hypoxic conditions. White bars with dashes denote normoxic conditions with addition of αPD-1 (B) or αTIGIT (C) and grey bars with dashes denote hypoxic conditions with αPD-1 (B) or αTIGIT (C). *p<0.05, **p<0.01, ***p<0.001, paired parametric *t*-test. Representative dot plots are shown in Fig. S1.

PD-1 blockade significantly decreased the percentage of early-stage apoptotic OE33 cells under hypoxia compared with untreated cells cultured under hypoxia (23.82 ± 3.2% vs. 28.02 ± 4.1%, *p* = 0.04), (Fig. 4**B.**). In addition, PD-1 blockade significantly decreased the percentage of early-stage apoptotic OE33 cells cultured under combined serum-deprivation with hypoxia compared with untreated OE33 cells cultured under these conditions (56.23 ± 2.9% vs. 60.27 ± 3.4%, *p* = 0.02), (Fig. 4**B.**). However, PD-1 blockade did not significantly affect the percentage of late-stage apoptotic OE33 cells cultured in cRPMI, nutrient-deprived conditions or hypoxia (Fig. 4**B.**).

Interestingly, PD-1 blockade under serum-deprivation significantly increased the percentage of early-stage apoptotic OE19 cells compared with untreated cells cultured under those conditions (40.20 ± 4.5% vs. 24.18 ± 3.8%, *p* = 0.003), (Fig. 4**B.**). However, under glucose-deprivation PD-1 blockade significantly decreased the percentage of early-stage apoptotic OE19 cells compared with untreated cells cultured under those conditions (28.14 ± 1.9% vs. 33.22 ± 1.2%, *p* = 0.01), (Fig. 4**B.**). Interestingly, under combined glucose and serum-deprivation in combination with hypoxia, PD-1 blockade significantly decreased the percentage of late-stage apoptotic OE19 cells compared with untreated cells cultured under those conditions (27.53 ± 1.3% vs. 53.48 ± 11.11%, *p* = 0.05), (Fig. 4**B.**).

TIGIT blockade significantly increased the percentage of late-stage apoptotic OE33 cells cultured under serum-deprivation compared with untreated cells cultured under those conditions (28.24 ± 6.0% vs. 17.9 ± 2.8%, *p* = 0.03), (Fig. 4**C.**). Similarly, TIGIT blockade significantly increased the percentage of late-stage apoptotic OE33 cells under glucose-deprived conditions compared with untreated cells cultured under those conditions (47.10 ± 1,4% vs. 31.72 ± 1.8%, *p* = 0.008), (Fig. 4**C.**). In contrast, TIGIT blockade under combined serum-deprivation in combination with hypoxia significantly decreased the percentage of early-stage apoptotic OE33 cells compared with untreated cells cultured under those conditions (40.38 ± 4.2% vs. 60.27 ± 3.4%, *p* = 0.001#), (Fig. 4**C.**). Subsequently, TIGIT blockade significantly increased the percentage of late-stage apoptotic OE33 cells cultured under combined serum-deprivation in combination with hypoxia compared with untreated cells cultured under those conditions (26.02 ± 2.5% vs. 22.52 ± 2.0%, *p* = 0.02), (Fig. 4**C.**).

Complementary findings were observed with the OE19 cell line. TIGIT blockade significantly increased the percentage of early-stage apoptotic OE19 cells under cRPMI conditions compared with untreated cells cultured under those conditions (29.32 ± 3.3% vs. 25.70 ± 2.6%, *p* = 0.05), (Fig. 4**C.**). TIGIT blockade significantly increased the percentage of early-stage apoptotic OE19 cells under serum-deprivation compared with untreated cells cultured under those conditions (40.20 ± 4.5% vs. 24.18 ± 3.8%, *p* = 0.003), (Fig. 4**C.**). TIGIT blockade significantly decreased the percentage of early-stage apoptotic OE19 cells and subsequently increased the percentage of late-stage apoptotic cells under glucose-deprivation compared with untreated cells cultured under those conditions (early-stage apoptotic cells:28.14 ± 1.9% vs. 33.22 ± 1.2%, *p* = 0.01, late-stage apoptotic cells: 43.60 ± 8.9% vs. 16.11 ± 2.7%, *p* = 0.04), (Fig. 4**C.**). TIGIT blockade also induced OE19 cell death under complete RPMI conditions demonstrated by a significant increase in the percentage of late-stage apoptotic cells (21.63 ± 0.8% vs. 15.75 ± 0.8%, *p*<0.0001), (Fig. 4**C.**). Similarly, TIGIT blockade significantly increased the percentage of late-stage apoptotic cells cultured under serum-deprivation, (26.17 ± 3.2% vs. 17.65 ± 3.3%, *p* = 0.02), (Fig. 4**C.**). TIGIT blockade significantly increased the percentage of late-stage apoptotic cells cultured under dual serum- and glucose-deprived conditions compared with untreated cells cultured under those conditions (39.3 ± 4.5% vs. 25.35 ± 2.7%, *p* = 0.004), (Fig. 4**C.**). Moreover, there was no significant effect of TIGIT blockade on the percentage of late-stage apoptotic OE19 cells cultured under hypoxia or combined hypoxia-nutrient deprivation (Fig. 4**C.**).

Overall, PD-1 blockade induced apoptosis under serum- and glucose-deprivation however, under hypoxia or combined serum- and glucose-deprivation with hypoxia PD-1 blockade conferred OAC cells with a survival advantage and decreased apoptosis and OAC cell death. Whereas, TIGIT blockade induced apoptosis and OAC cell death under nutrient-deprivation and hypoxia.

### PD-1 blockade and TIGIT blockade decreases the levels of anti-apoptotic proteins Bcl-2 and Bcl-xL in OAC cells

**Fig. 5:**
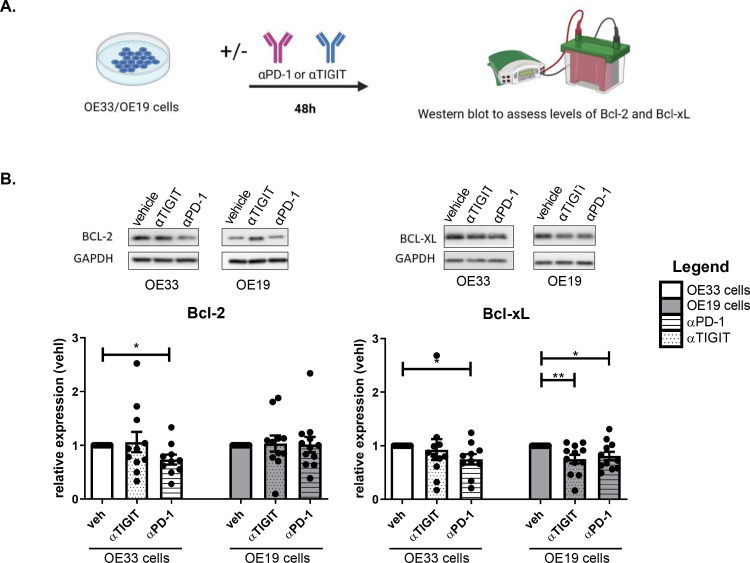
TIGIT blockade decreases the expression of anti-apoptotic factor Bcl-xL in OAC cells in vitro. (A) Schematic workflow. OE33 cells and OE19 cells were treated with αPD-1 (pembrolizumab, 10 μg/ml) or αTIGIT (10 μg/ml) for 48 h. (B) Expression levels of Bcl-2 and Bcl-xL were determined by western blot. β-actin or GAPDH were used as housekeeping controls to normalise protein expression. Representative blot images of OE19 cell line. Data expressed as ± SEM relative to vehicle (veh) control. Pooled analysis for 11 independent biological replicates carried out in singlet technical replicates for the OE33 cell line and pooled analysis for 12 independent biological replicates carried out in singlet technical replicates for the OE19 cell line. *p<0.05, paired parametric *t*-test.

To further elucidate the effect of inhibiting PD-1 and TIGIT tumour cell intrinsic signalling on pro-survival pathways in OAC cells, the levels of anti-apoptotic proteins Bcl-2 and Bcl-xL were assessed following single agent PD-1 and TIGIT blockade in OE33 and OE19 cells (Fig. 5**.**). PD-1 blockade significantly decreased the levels of Bcl-2 in OE33 cells compared with untreated cells (αPD-1:0.73 ± 0.1% vs. veh:1.0 ± 0.0%, *p* = 0.01) (Fig. 5**B.**). In addition, PD-1 blockade significantly decreased Bcl-xL levels in OE33 cells compared with untreated cells (αPD-1:0.74 ± 0.1% vs. veh:1.0 ± 0.0%, *p* = 0.02), (Fig. 5**B.**). However, ICB did not significantly affect the levels of Bcl-2 in the OE19 cell line. Single agent PD-1 blockade and TIGIT blockade significantly decreased the levels of Bcl-xL in OE19 cells compared with untreated cells (αPD-1:0.80 ± 0.1%, αTIGIT:0.74 ± 0.1% vs. veh:1.0 ± 0.0%, *p* = 0.03 and *p* = 0.009, respectively), (Fig. 5**B.**).

Overall, ICB significantly reduced the levels of anti-apoptotic proteins in OAC cells, however, these effects were dependent on the particular cell line, ICB and anti-apoptotic factor under question. In summary, the levels of Bcl-xL were more substantially affected by ICB than the levels of Bcl-2 across the two OAC cell lines. PD-1 blockade significantly decreased Bcl-xL levels in the OE33 and OE19 cell line, whereas TIGIT blockade only decreased Bcl-xL in the OE19 cells.

### Single agent PD-1 blockade and TIGIT blockade in OAC cells differentially alters tumour cell metabolism

**Fig. 6:**
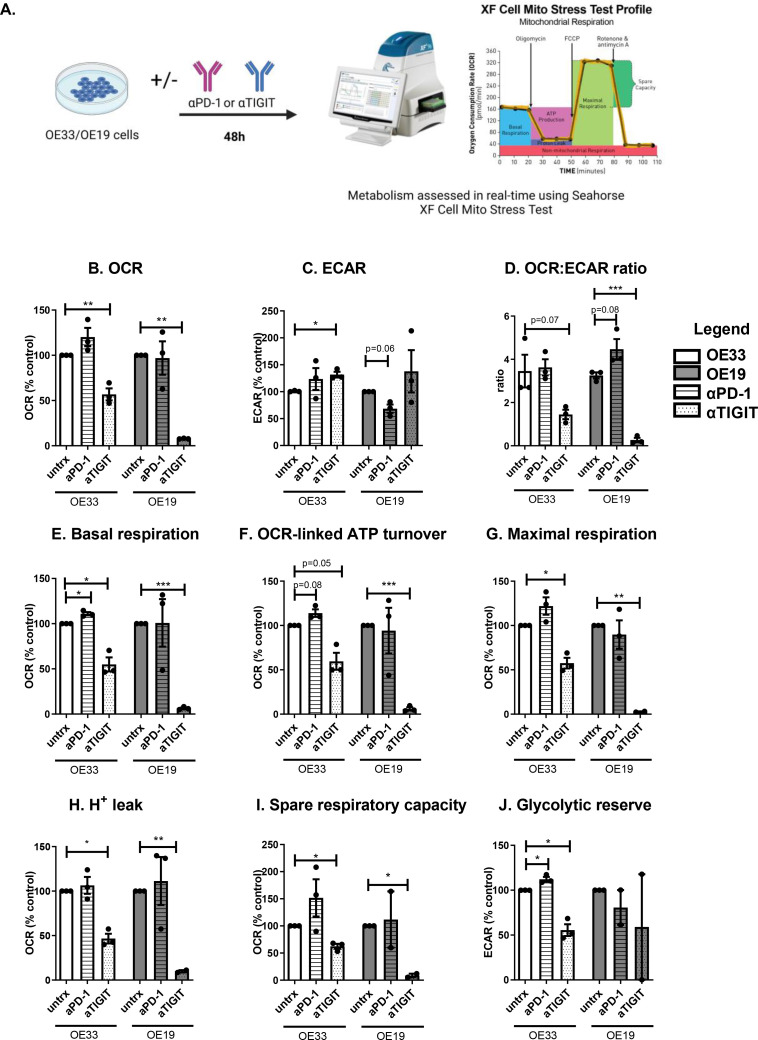
PD-1 blockade increases basal respiration and glycolytic reserve whereas TIGIT blockade increases ECAR and decreases OCR in OE33 cells. OE33 and OE19 cells were treated with and without αPD-1 (pembrolizumab, 10 μg/ml) or αTIGIT monoclonal antibody (10 μg/ml) for 48 h. OCR, a measure of oxidative phosphorylation and ECAR, a measure of glycolysis was assessed in real-time using the Seahorse Biosciences XFe24 Analyser. (A) Schematic workflow showing the Seahorse XF Mito Stress Test that was used. A range of metabolic parameters were then calculated from the mito stress test profile using Seahorse technology including: OCR (B.), ECAR (C.), OCR:ECAR ratio (D.), basal respiration (E.), OCR-linked ATP turnover (F.), maximal respiration (G.), *H*^+^ leak (H.), spare respiratory capacity (I.) and glycolytic reserve (J.) All data expressed as mean ± SEM. Data expressed relative to untreated cells set to 100% (B-J). Pooled analysis for 3 independent biological replicates conducted using triplicate technical replicates. Paired parametric *t*-test, *p<0.05, **p<0.01.

Given that the findings from this study demonstrated that glucose deprivation and hypoxia significantly upregulate PD-1 and TIGIT on the surface of OAC cells in vitro and that these conditions have substantial effects on the metabolism of cancer cells, we sought to investigate if PD-1 blockade or TIGIT blockade might alter the metabolism of OAC cells. Seahorse Biosciences XFe24 Extracellular Flux Analyser was used to measure changes in metabolism in real time (Fig. 6**A.**).

TIGIT blockade significantly increased ECAR, a measure of glycolysis in OE33 cells compared with vehicle control (veh:100.8 ± 0.7% vs. αTIGIT:131.7 ± 5.3%, *p* = 0.03, respectively), (Fig. 6**B.**). In contrast, TIGIT blockade significantly decreased OCR a measure of oxidative phosphorylation in OE33 cells and OE19 cells compared with vehicle control (OE33 cells: veh:100 ± 0.0% vs. αTIGIT:56.79 ± 6.7% and OE19 cells:veh:100.0 ± 0.0% vs. αTIGIT:7.88 ± 0.22%, *p* = 0.02 and *p*<0.0001, respectively), (Fig. 6**B.**). TIGIT blockade also significantly decreased the OCR:ECAR ratio in OE19 cells compared with the vehicle control (veh:3.25 ± 0.14 vs. αTIGIT:0.26 ± 0.1, *p* = 0.001), (Fig. 6**D.**). PD-1 blockade significantly increased basal respiration in OE33 cells compared with the vehicle control (veh:100 ± 0.0% vs. αPD-1:111.0 ± 1.9%, *p* = 0.02), (Fig. 6**E.**). Contrastingly, TIGIT blockade significantly reduced basal respiration in OE33 cells (veh:100 ± 0.0% vs. αTIGIT:54.93 ± 7.7%, *p* = 0.02), (Fig. 6**E.**). Similarly, TIGIT blockade significantly reduced the basal respiration in OE19 cells compared with the vehicle control (veh:100 ± 0.0% vs. αTIGIT:6.37 ± 1.0%, *p*<0.0001). Furthermore, TIGIT blockade significantly decreased OCR-linked ATP turnover in OE33 cells and OE19 cells compared with vehicle control (OE33 cells: veh:100 ± 0.0% vs. αTIGIT:59.6 ± 9.5% and OE19 cells:veh:100.0 ± 0.0% vs. αTIGIT:5.9 ± 1.8%, *p* = 0.05 and *p* = 0.0004, respectively), (Fig. 6**F.**). Maximal respiration is also significantly reduced following TIGIT blockade compared with vehicle control in both OE19 cells and OE33 cells (OE33 cells: veh:100 ± 0.0% vs αTIGIT 57.57 ± 5.9%. OE19 cells: veh:100 ± 0.0% vs. αTIGIT:2.76 ± 0.45%, *p* = 0.01 and *p* = 0.003, respectively), (Fig. 6**G.**). TIGIT blockade significantly decreased proton leak in both OE33 and OE19 cells compared with vehicle control (OE33 cells: veh:100 ± 0.0% vs. αTIGIT:46.63 ± 5.5% and OE19 cells:veh:100.0 ± 0.0% vs. αTIGIT:9.9 ± 1.3%, *p* = 0.01 and *p* = 0.009, respectively), (Fig. 6**H.**). Furthermore, TIGIT blockade significantly reduced the spare respiratory capacity in OE33 cells and OE19 cells compared with the vehicle control (OE33 cells: veh:100 ± 0.0% vs. αTIGIT: 62.35 ± 4.6% and OE19 cells: veh:100.0 ± 0.0% vs. αTIGIT: 8.65 ± 3.3%, *p* = 0.01 and *p* = 0.02, respectively), (Fig. 6**I.**). PD-1 blockade and TIGIT blockade significantly increased and decreased the glycolytic reserve in OE33 cells compared with vehicle control, respectively (veh:100 ± 0.0% vs. αPD-1: 112.1 ± 2.6%, αTIGIT: 55.6 ± 6.6%, *p* = 0.04 and *p* = 0.02 respectively), (Fig. 6**J.**).

Overall, PD-1 blockade enhanced basal respiration and glycolytic reserve in OE33 cells compared with the vehicle control. Whereas, TIGIT blockade increases ECAR yet decreased several metabolic parameters in OE33 cells including oxidative phosphorylation, basal respiration, OCR-linked ATP turnover, maximal respiration, proton leak, spare respiratory capacity and glycolytic reserve.

**Fig. 7:**
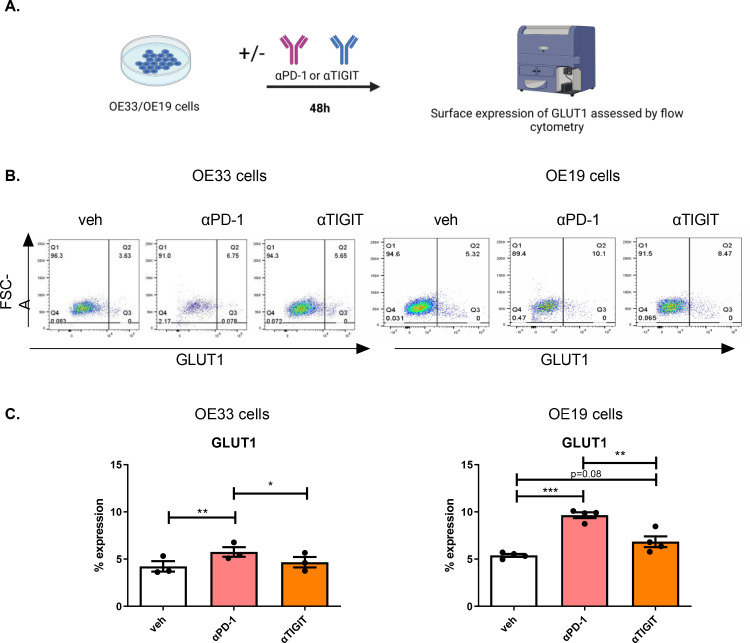
Pembrolizumab and αTIGIT significantly increase the percentage of OE19 cells expressing GLUT1. (A) Schematic workflow. OE33 and OE19 cells were treated with vehicle (veh) control, αPD-1 (pembrolizumab, 10 μg/ml) or αTIGIT monoclonal antibody (10 μg/ml) for 48 h. The percentage of live OAC cells expressing GLUT1 was determined by flow cytometry. (B) Representative dot plots showing effect of vehicle control, PD-1 blockade or TIGIT blockade on the percentage of GLUT1 positive OE33 and OE19 cells. (C) Depicts the effect of PD-1 blockade or TIGIT blockade on the surface expression of GLUT1 on OE33 and OE19 cells. Pooled analysis for 4 biological replicates carried out in singlet technical replicates were included for both the OE33 and OE19 cell lines. Vehicle control includes 2% saline. Paired parametric *t*-test. *p<0.05, **p<0.01 and ***p<0.001. Data expressed as ± SEM.

The findings from this study demonstrated that PD-1 blockade significantly enhanced glycolytic reserve in OAC cells whereas, TIGIT blockade significantly enhanced a glycolytic phenotype yet decreased glycolytic reserve in OAC cells. Therefore, we sought to investigate if PD-1 blockade or TIGIT blockade affected the surface expression of GLUT1 by OAC cells, which is an important transporter of glucose from the external TME into OAC cells (Fig. 7**C.**). Interestingly, PD-1 blockade significantly upregulated the surface expression of GLUT1 on the surface of OE33 cells and OE19 cells compared with untreated cells (OE33 cells: 5.76 ± 0.5% vs. 4.23 ± 0.5%, *p* = 0.008 and OE19 cells: 9.65 ± 0.3% vs. 5.39 ± 0.2, *p* = 0.0003), (Fig. 7**C.**). There was a trend toward an increase in the surface expression of GLUT1 on the surface of OE19 cells following TIGIT blockade compared with untreated cells (6.84 ± 0.6% vs. 5.39 ± 0.2, *p* = 0.08) (Fig. 7**C.**). Overall, PD-1 blockade had the greatest effect at upregulating GLUT1 on the surface of OAC cells in vitro.

### Inhibition of oxidative phosphorylation and glycolysis in OAC cells differentially alters the surface expression of tumour-intrinsic PD-1 and TIGIT

**Fig. 8:**
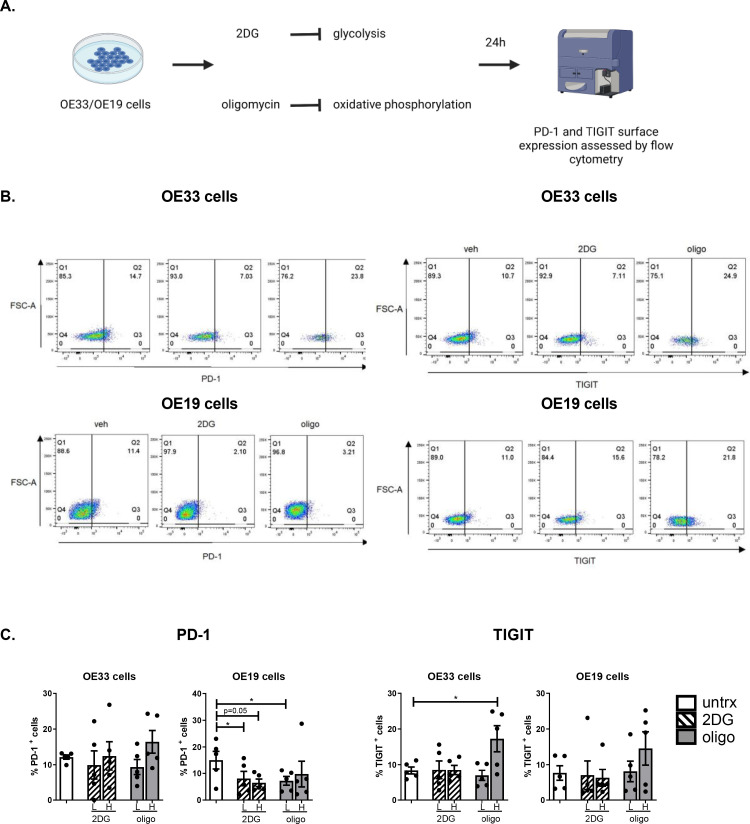
Inhibition of glycolysis and oxidative phosphorylation decreases PD-1 on the surface of a subpopulation of OE19 cells whereas, inhibition of oxidative phosphorylation increases TIGIT on the surface of a subpopulation of OE33 cells. (A) Schematic workflow. OE33 and OE19 cells were treated with vehicle control or 2DG (low dose (L): 10 mM and high dose (H): 20 mM) or oligomycin (low dose: 3 μM and high dose: 6 μM) for 24 h and the percentage of viable cells expressing PD-1 and TIGIT was determined by flow cytometry (C). Pooled analysis for 5 independent biological replicates carried out in technical singlets were conducted. (B) Representative dot plots showing effect of vehicle, high dose 2DG and high dose oligomycin on PD-1 and TIGIT expression on the surface of OE19 cells and OE33 cells, respectively. Paired parametric *t*-test. *p<0.05. Abbreviations: 2DG, 2-deoxy-d-glucose; H, high dose; L, low dose; oligo, oligomycin.

**Fig. 9:**
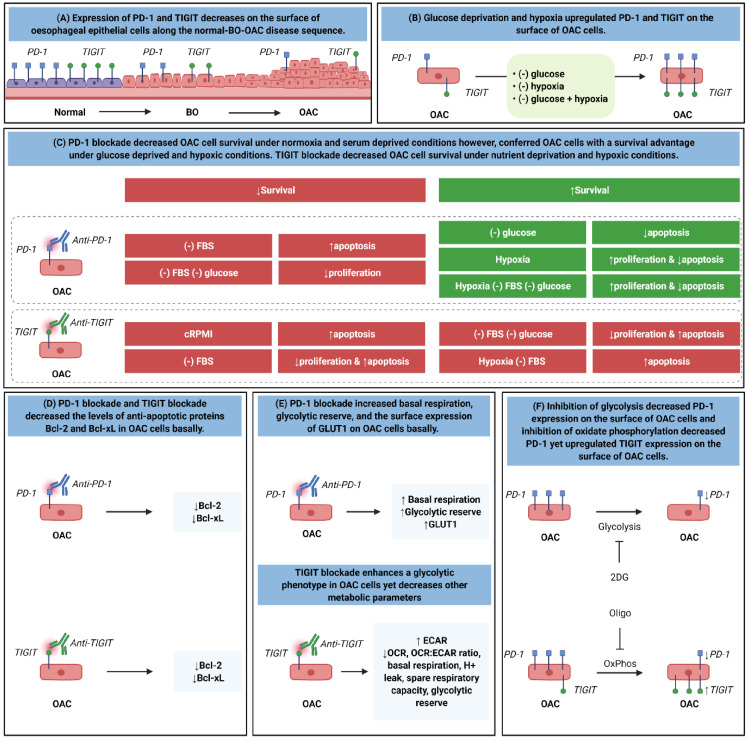
Graphical summary of the key findings from the study. (A) Expression of PD-1 and TIGIT decreases on the surface of oesophageal epithelial cells along the normal-BO—OAC disease sequence. (B) Glucose deprivation and hypoxia upregulated PD-1 and TIGIT on the surface of OAC cells. (C) PD-1 blockade decreased OAC cell survival under normoxia and serum deprived conditions however, conferred OAC cells with a survival advantage under glucose deprived and hypoxic conditions. TIGIT blockade decreased OAC cell survival under nutrient deprivation and hypoxic conditions. (D) PD-1 blockade and TIGIT blockade decreased the levels of anti-apoptotic proteins Bcl-2 and Bcl-xL in OAC cells basally. E. PD-1 blockade increased basal respiration, glycolytic reserve, and the surface expression of GLUT1 on the surface of a subpopulation of OAC cells basally. TIGIT blockade enhanced a glycolytic phenotype in OAC cells yet decreased other metabolic parameters basally. (F) Inhibition of glycolysis decreased PD-1 expression on the surface of a subpopulation of OAC cells and inhibition of oxidate phosphorylation decreased PD-1 yet upregulated TIGIT expression on the surface of a subpopulation of OAC cells.

The findings from this study have demonstrated that both PD-1 and TIGIT blockade significantly alter OAC cell metabolism, we therefore sought to investigate if inhibiting oxidative phosphorylation and glycolysis in OAC cells might affect the expression of PD-1 or TIGIT on the surface of OAC cells in vitro (Fig. 8**.**). Inhibition of glycolysis or oxidative metabolism had no significant effect on the expression of PD-1 in OE33 cells compared with the vehicle control (Fig. 8**C.**). However, inhibition of glycolysis with a low dose of 2DG significantly reduced PD-1 expression on the surface of OE19 cells (8.06 ± 2.7 vs. 15.05 ± 3.2%, *p* = 0.03), (Fig. 8**C.**). Additionally, inhibition of oxidative phosphorylation using a low dose of oligomycin significantly reduced the expression of PD-1 on the surface of OE19 cells (7.21 ± 1.7 vs. 15.05 ± 3.2%, *p* = 0.02), (Fig. 8**C.**). In contrast, inhibition of oxidative phosphorylation significantly upregulated TIGIT on the surface of OE33 cells compared with the vehicle control (17.26 ± 3.7 vs. 8.33 ± 0.1%, *p* = 0.04), (Fig. 8**C.**). Inhibition of glycolysis or oxidative phosphorylation had no significant effect on the surface expression of TIGIT in OE19 cells (Fig. 8**C.**) (Fig. 9).

Overall, inhibition of oxidative phosphorylation and glycolysis decreased the surface expression of PD-1 on OAC cells and inhibition of oxidative phosphorylation significantly upregulated TIGIT on the surface of OAC cells.

## Discussion

Given the key role of ICs in maintaining immune tolerance and inhibition of autoimmunity [22] the observed decrease in the expression of PD-1 and TIGIT on the surface of oesophageal epithelial cells across the normal-BO—OAC disease sequence in this study might reflect a loss of homoeostasis and immune tolerance with disease development and progression. A wide range of ICs exist beyond the well-studied PD-1 and CTLA-4 IC receptors and it is hypothesised that specific ICs have unique roles in maintaining immune tolerance at tissue specific sites [23]. Therefore, these findings suggest that PD-1 and TIGIT IC receptors may play an important role in the oesophagus for maintaining immune tolerance at this tissue specific site. Inflammation plays an important role in the progression of BO to OAC [24] therefore, loss of PD-1 and TIGIT may play a key role in the uncontrolled amplitude and duration of inflammatory responses in the oesophagus. Ligation of PD-1 and TIGIT to their respective ligands PD-L1 [25] and poliovirus receptor [26] on the surface of dendritic cells promotes a regulatory dendritic cell phenotype that consequently dampens inflammatory responses. Similarly, ligation of PD-L1 to PD-1 on the surface of T cells promotes a regulatory phenotype [27]. In addition, ligation of TIGIT to poliovirus receptor on the surface of macrophages induces an M2 anti-inflammatory phenotype, which would help resolve pathogenic inflammation [28]. Therefore, the decrease in PD-1 and TIGIT expression in the early stages of metaplastic disease on the surface of oesophageal epithelial cells may facilitate the generation of pathogenic inflammation that plays a key role in promoting the conversion of BO to OAC.

However, it is important to note that although PD-1 and TIGIT expression on the surface of OAC cells decreases along the normal-BO—OAC disease sequence, stressful conditions that recapitulate characteristic features of the TME such as hypoxia [29] and glucose deprivation [30] upregulated PD-1 and TIGIT on the surface of OAC cells. Under these hostile conditions, PD-1 and TIGIT might play a unique role in promoting the survival of OAC cells within the TME.

In this study the effect of removing glucose was the same as hypoxia treatment or the combination of both conditions in OE33 cells. However, the contrary was observed in the OE19 cells where the combined conditions had a greater effect than the single treatments on PD-1 and TIGIT expression. This may be a suggest that the OE19 cell line may be more resistant to the cellular stress induced by these harsh treatments compared with the OE33 cell line as PD-1 and TIGIT upregulation may be an attempt by the tumour cells to confer a survival advantage.

Two cell lines with different doubling times were used to carry out these experiments to try an encapsulate the heterogeneity of human tumours in vivo (OE33 cells: 33 h and OE19 cells: 41 h). The cytotoxicity and resulting cell stress is often dependent on how quickly the cell proliferates and passes through the cell cycle. Therefore, single and combined harsh conditions may have reached a threshold of cellular stress within the remaining viable OE33 cell population and in parallel upregulated PD-1 and TIGIT to a threshold of expression. In contrast, the OE19 cells grow more slowly and therefore, may not have acquired the same level of cellular stress within the viable population of cells and this may explain why differences can be seen between treatment with single and combined harsh conditions. Using longer culture conditions the same level of PD-1 and TIGIT expression may be observed in the OE19 cell line as well using single and combined treatments. Further differences also exist between the two cell lines used in this study. These cell lines were chosen based on these differing characteristics to more holistically recapitulate the different tumour cells that populate different patient's tumours. These two cell lines were isolated from patients of opposite gender whose tumours were of different pathological stage, differentiation status, different sites of origin within the oesophagus and with distinct functional mutations in p53 protein. The OE19 cell line was established from an adenocarcinoma of gastric cardia/oesophageal gastric junction of a 72 year old male patient with a pathological stage III and showed moderate differentiation. Whereas, the OE33 cell line was established from the adenocarcinoma of the lower oesophagus arising from the pre-malignant condition Barrett's metaplasia from a 73 year old female patient with a pathological stage IIA and showed poor differentiation. Both cell lines possess distinct mutations in the p53 gene. OE33 cells have a point mutation in exon 5 (c.404G>*A*, p.C135Y), which abolishes the p53 transactivation activity as well as the growth suppressive activity of the mutated protein and has a dominant negative effect on wild type p53^3,4^. Additionally, OE19 cells exhibit a mutation in exon 9 (c.928_930insA, p.N310fs26X), within the flexible linker, which connects the p53 core domain with the tetramerization domain resulting in a stop codon within the tetramerization domain and most likely inactivates p53 oligomerization [5]. However, this mutation is insufficient to fully abolish p53 tumour suppressive function and p53 monomer mutants with retention of transcriptional activity still being observed [6]. In contrast to the OE33 cells which possess a mutation that abolishes p53 growth suppressive activity, OE19 cells still exhibit a functional mutated p53 protein, which is strongly expressed as a truncated protein and clearly accumulates in OE19 cell nuclei [5]. Collectively, these reasons may all contribute to differences observed between the OE33 and OE19 cells. Further research is required to fully elucidate if PD-1 or TIGIT blockade hold real potential as new treatment options and using more complex tumour models such as tumour organoids and patient-derived xenograft models to encapsulate the heterogeneous landscape of OAC will be essential.

These findings contrast those from a study in pancreatic ductal adenocarcinoma, which demonstrated that PD-1 expression was significantly upregulated along the normal-pre-cancerous-pancreatic ductal adenocarcinoma disease sequence [20]. These differences may be explained by the distinct differences in cell types and tissue types between oesophageal epithelial cells and pancreatic epithelial cells highlighting tissue specific roles for distinct ICs. However, characteristic conditions of the TME that recapitulate the hostile microenvironment, such as hypoxia and glucose deprivation induced PD-1 and TIGIT upregulation on the surface of OAC cells that were similar to the levels of expression on HET-1A and QH cells.

This study demonstrates that PD1 expression decreases specifically on the surface of oesophageal epithelial cells across the disease progression sequence from normal-BO—OAC. In contrast, the RNA expression analysis of the entire tissue which encapsulates all cells within the tissue such as oesophageal epithelial cells, immune cells, stromal cells and presented an opposite view whereby PD-1 did not significantly decrease along the normal-BO—OAC disease sequence. As the tissue sample contains many different types of immune cells across the normal-BO—OAC disease sequence the decrease in PD-1 expression on the surface of oesophageal epithelial cells may be masked by immune cells which upregulate PD-1 on their surface as a result of immune exhaustion, which would be expected in the pre-malignant condition BO and OAC [1,2].

PD-1 and TIGIT are well-known for their role in dampening anti-tumour immunity and promoting tumour progression [31]. However, these findings identify novel immune-independent functions for PD-1 and TIGIT tumour cell intrinsic signalling in promoting OAC cell survival. Subsequent inhibition of PD-1 and TIGIT under complete nutrient conditions decreased the levels of anti-apoptotic proteins, decreased cell proliferation and induced apoptosis in OAC cells. A complementary study by Liu et al., demonstrated that PD-1-intrinsic expression in 5-FU resistant gastric cancer cells promoted resistance via upregulation of Bcl-2^15^. Similar studies in other cancer types highlighted a similar role for PD-1 intrinsic signalling in pancreatic ductal adenocarcinoma [20], melanoma [32], thyroid [33], liver [34], and head and neck cancer cells, whereby PD-1 signalling increased tumour cell proliferation and viability. In contrast, PD-1 blockade in non-small cell lung cancer cells promoted cancer cell proliferation, highlighting the context-dependent role of PD-1-intrinsic signalling in cancer cells as either a tumour promoter or tumour suppressor [35].

Interestingly, PD-1 blockade under glucose deprivation or hypoxia enhanced OAC cell proliferation and protected against apoptotic-induced cell death. This might be explained by a deeper insight into the mechanistic functions of PD-1 signalling in OAC cells provided in this study. Interestingly, this study uncovered a mechanistic rationale that may explain why PD-1 inhibition under glucose deprivation or hypoxia is conferring OAC cells with a survival advantage. PD-1 inhibition enhanced a range of metabolic parameters in OAC cells including basal respiration and glycolytic reserve, which may be enhancing a metabolic phenotype in OAC cells potentially enabling OAC cells to better tolerate and adapt to the harsh glucose deprived and hypoxic conditions found within the TME in OAC. Given that tumour cells rely on glycolysis for the production of ATP under hypoxia, the findings from this study demonstrate that inhibition of PD-1 intrinsic signalling in OAC cells upregulates GLUT1 on the surface of a subpopulation of OAC cells, further supporting the hypothesis that inhibition of PD-1 under glucose-deprivation or hypoxia in OAC cells is enhancing glycolysis and subsequent survival under these harsh conditions. A complementary study demonstrated that PD-1 inhibition in a mouse model of B16F10 melanoma resulted in an increase in GLUT1 expression and subsequent increase in the uptake of glucose analogue [^18^F]FDG by cancer cells [36]. Collectively, these findings may suggest that PD-1 inhibitors may promote OAC cell survival under hypoxia or glucose deprived microenvironments within the tumour. The lack of efficacy of PD-1 inhibitors in hypoxic tumours has been attributed to hypoxia-induced immunosuppression [37] however, these findings suggest an immune-independent mechanistic rationale for the lack of efficacy of PD-1 inhibitors in hypoxic tumours.

Notably, although we did observe a small but significant increase in GLUT1 expression in OE19 cells, this did not translate or correspond with an increase in glycolytic reserve. This suggests that an increase in glucose uptake was not sufficient to increase the glycolytic reserve in the OE19 cells, importantly this underpins the necessity to carry out functional experiments such as real-time metabolic profiling to really discern what effects drugs are having on metabolism as an increase in glucose influx pumps is not a surrogate marker for an increase in glycolysis. While the cell may be attempting to increase their glucose intake, this does not indicate that the cell has also increased glycolysis.

However, this phenomenon was specific to the PD-1 IC receptor pathway as inhibition of TIGIT intrinsic signalling in OAC cells under serum deprivation or glucose deprivation or hypoxic conditions substantially decreased OAC cell survival. Although TIGIT has been identified on the surface of colorectal cancer cells [9] a role for TIGIT in promoting cancer cell survival via tumour cell intrinsic signalling has not been identified in previous studies.

Given that PD-1 and TIGIT inhibition significantly altered metabolism in OAC cells and that inhibition of oxidative phosphorylation or glycolysis significantly altered PD-1 and TIGIT expression on the surface of a subpopulation of OAC cells, this further highlights a role for PD-1 and TIGIT tumour cell intrinsic signalling in regulating OAC cell metabolism and may potentially suggest the existence of a positive/negative feedback loop between the expression of these ICs and regulation of metabolism in OAC cells. Further studies are necessary to determine if PD-1 and TIGIT tumour cell intrinsic signalling may regulate OAC cell metabolism.

The fact that inhibiting glycolysis or oxidative phosphorylation decreased PD-1 expression in OAC cells may suggest that PD-1 signalling may interconnect with both forms of metabolism. Metabolic pathways do not function separately or independently in cells, it is much more complex, these pathways often feed into each other's cycles, and one cell will often rely on multiple forms of metabolism. In this study we demonstrated that inhibition of PD-1 signalling increased OCR and glycolytic reserve suggesting both glycolysis and oxidative phosphorylation are affected by PD-1 intracellular signalling in OAC cells and it is not surprising that inhibiting both oxidative phosphorylation or glycolysis would affect PD-1 expression in turn. Taken together, this suggests that a complex feedback loop exists between PD-1 signalling, oxidative phosphorylation and glycolysis. However, it is unclear what the implications are and further research is required to elucidate the intricate complexity of PD-1 signalling and how it interconnects with different metabolic pathways in OAC cells.

Although PD-1 and TIGIT blockade had a minimal but significant effect on OAC cell viability across different conditions, which have very complex and differential effects on OAC cell phenotypes and survival, this should not necessarily diminish the potential clinical relevance or biological significance of these findings. A small decrease in viability may reflect a reduction in the survival of a subpopulation of cells which may be more therapy refractory and hence a reduction in survival factors within this population would be of significant clinical and biological significance which certainly warrants further investigation.

To conclude, these findings implicate a potential role for PD-1 and TIGIT for maintaining immune tolerance in the oesophagus, suggesting that these ICs may be appropriate ICs to target in oesophageal cancers. In addition, inhibition of TIGIT signalling on the surface of OAC cells under full nutrient conditions, nutrient deprivation and hypoxia consistently decreased OAC cell survival and induced OAC cell death. However, PD-1 inhibition under normoxia reduced OAC cell survival but under glucose deprivation and hypoxia inhibition of PD-1 intrinsic signalling in OAC cells enhanced OAC cell survival. Collectively, these findings may suggest a novel hypothesis that TIGIT may be a better IC to target in OAC than PD-1 to reinvigorate anti-tumour immunity but to also reduce the survival of OAC cells under the nutrient-deprived and hypoxic conditions of the hostile TME. Alternatively, combining TIGIT immune checkpoint blockade with PD-1 blockade may counteract any potential pro-survival effects of PD-1 blockade in tumour cells under hypoxic and nutrient deprived conditions. Additional research using both immune competent and immunodeficient models will be necessary to definitively answer this timely research question. It will also be critical to carry out further studies to interrogate which ICs are the most appropriate to target in OAC patients to not only enhance anti-tumour immunity but to also directly inhibit the pro-survival immune-independent functions of ICs in OAC cells. Clinical trials will be useful in this regard to identify the optimal immune checkpoint to target in OAC patients.

## Declaration of Competing Interest

The authors declare that there is no conflict of interest that could be perceived as prejudicing the impartiality of the research reported.

## Appendix Supplementary materials

Image, application 1
