# Supplementary material for: PD-1 and TIGIT blockade differentially affect tumour cell survival under hypoxia and glucose deprived conditions in oesophageal adenocarcinoma; implications for overcoming resistance to PD-1 blockade in hypoxic tumours
Source: Transl Oncol. 2022 Mar 1;19:101381. doi: 10.1016/j.tranon.2022.101381 (PMC8894275; doi:10.1016/j.tranon.2022.101381)
Supplement: Supplementary file 1 [file mmc1.docx]

**Fig. S1. Representative dot plots demonstrating that PD-1 blockade decreases OAC cell death under hypoxia and TIGIT blockade induces OAC cell death basally and under nutrient deprivation.** (A) and (B) depicts gating strategies for gating on viable OE33 and viable OE19 cells, respectively. (C) Representative dot plots shown for OE19 cells cultured under in complete media, serum deprived (no FBS), glucose deprived, combined serum deprived-glucose deprived, hypoxia and combined serum deprived-hypoxia, combined glucose deprived-hypoxia and combined serum deprived-glucose deprived-hypoxia for 48h in the absence or presence of αPD-1 (pembrolizumab, 10 μg/ml) or αTIGIT monoclonal antibody (10 μg/ml). Viability was determined by annexin V propidium iodide assay by flow cytometry. Viable cells (AV^-^PI^-^), early-stage apoptotic cells (AV^+^PI^-^), late-stage apoptotic cells (AV^+^PI^+^) and necrotic cells (AV^-^PI^+^) were characterised.

Supplemental****

**Fig. S1. Representative dot plots demonstrating that PD-1 blockade decreases OAC cell death under hypoxia and TIGIT blockade induces OAC cell death basally and under nutrient deprivation.** (A) and (B) depicts gating strategies for gating on viable OE33 and viable OE19 cells, respectively. (C) Representative dot plots shown for OE19 cells cultured under in complete media, serum deprived (no FBS), glucose deprived, combined serum deprived-glucose deprived, hypoxia and combined serum deprived-hypoxia, combined glucose deprived-hypoxia and combined serum deprived-glucose deprived-hypoxia for 48h in the absence or presence of αPD-1 (pembrolizumab, 10 μg/ml) or αTIGIT monoclonal antibody (10 μg/ml). Viability was determined by annexin V propidium iodide assay by flow cytometry. Viable cells (AV^-^PI^-^), early-stage apoptotic cells (AV^+^PI^-^), late-stage apoptotic cells (AV^+^PI^+^) and necrotic cells (AV^-^PI^+^) were characterised.
